# The health service capacity of primary health care in West China: different perspectives of physicians and their patients

**DOI:** 10.1186/s12913-019-3964-x

**Published:** 2019-02-28

**Authors:** Wenjuan Tao, Wenqi Zeng, Ling Yan, Huazhen Yang, Jin Wen, Weimin Li

**Affiliations:** 1 Institute of Hospital Management, West China Hospital, Sichuan University, Guo Xue Xiang 37, 610041 Chengdu, People’s Republic of China; 2 West China School of Public Health, Sichuan University, Chengdu, Sichuan China; 3 Department of Respiratory Medicine, West China Hospital of Sichuan University, Chengdu, Sichuan China

**Keywords:** Health service capacity, Primary care, QUALICOPC

## Abstract

**Background:**

Many countries, including China, have identified the primary health care system as a reform priority. The purpose of this study is to compare the perceived service capacity of primary care from the perspectives of physicians and their patients in Sichuan province of China.

**Methods:**

A cross-sectional survey was conducted through Quality and Costs of Primary Care (QUALICOPC) questionnaires. A representative sample of 319 primary care physicians and 641 patients in 48 primary healthcare settings were recruited to take part in the study.

**Results:**

Physicians perceived equity of care the best, while quality of care was rated the highest from the perspective of patients. They both regarded coordination as the weakest dimension of primary care service capacity.

**Conclusions:**

Although primary health care reform may have been effective in helping patients acquire better primary care services, our results suggest that coordination is still perceived to be problematic for both physicians and patients. Improving the coordination of care has to be one of the main goals in the future primary care reforms in China.

**Electronic supplementary material:**

The online version of this article (10.1186/s12913-019-3964-x) contains supplementary material, which is available to authorized users.

## Background

Many countries have identified the primary health care system as a priority for reform [1–3]. Primary care (PC) was identified by the Alma-Ata declaration as the foundation for integrating all health and social services to improve health outcomes, and the key to sustainable, accessible, and equitable health systems [4]. In China, a new health care reform was initiated in 2009, focusing on primary care (e.g., instituting universal health insurance coverage, a basic public health service program, and a national essential drug system) [5]. In 2014, the government implemented a hierarchical medical system (involving primary diagnoses at primary health care institutions and two-way referrals among different levels of hospitals), to strengthen the service capabilities of primary health care and increase reliance upon primary health care services [6].

Evidence from previous studies using administrative data has indicated a significant impact on primary care services since the 2009 reform, e.g., minimum subsidies per capita for basic public health service tripled from 2009 to 2016 [7], and an overall reduction in average prescription costs for patients [8]. However, the government’s efforts were not completely successful. People still seek care at relatively high-level hospitals for treatment, which leads to overcrowding in large hospitals and high healthcare expenditures [9, 10]. Therefore, there is a pressing need to comprehensively evaluate the impact of the 2009 reform on primary health care services in China.

The strength of a country’s primary care system depends on multiple dimensions of primary care impacts in the context of its health care system [11]. Previous Chinese studies mainly evaluated the primary care service on one dimension, such as equity [12], satisfaction [13] and continuity [14]. Some comprehensive primary care studies have been performed, for example, using surveys such as the Primary Care Assessment Tool (PCAT) [15, 16]. However, these assessments mainly focused on the perceptions of patients, and there is limited research exploring other perspectives, especially primary care physicians. Based on stakeholder theory, evaluating primary care should consider the perspectives of: patients (service users), health care professionals (service providers), and administrators (managers) [17, 18]. It is important to survey primary care physicians because they are the main providers of care in this reform [19]. Furthermore, evaluating primary care from multiple levels better reveals the relationships between the different levels of and provides insight into various stakeholder viewpoints.

The Quality and Costs of Primary Care (QUALICOPC) is an international study of primary care systems designed to understand how patients perceive the quality of primary care, how providers provide services, and overall health outcomes of primary care in 34 countries worldwide [20]. QUALICOPC questionnaires are developed to make a comprehensive analysis of primary care (PC). Evaluation of the service capacity of the PC system includes three levels: structural level (governance, economic conditions, and workforce development), process level (access, continuity of care, coordination of care, and comprehensiveness of care) and outcome level (quality of care, efficiency of care, and equity in health) [18, 19].

The aim of our study was to comprehensively evaluate the primary health care service capacity in China by investigating primary care physicians’ and their patients’ perceptions using the European QUALICOPC protocol. We compared and analyzed similarities and differences between the perspectives of physicians and their patients. Our goal is to contribute evidence for the improvement of Chinese health policy.

## Methods

### Setting and sampling

A cross-sectional survey was conducted in Sichuan province, which is located in the west of China. Sichuan province has a population of 82.62 million and the largest number of primary care institutions of all administrative divisions in China [21]. Multi-stage random cluster sampling was used to make the sample representative. We divided a total of 21 cities in Sichuan province into three levels according to the per capita Gross Regional Product (GRP). Two cities were randomly selected from each level: high-GRP areas (Chengdu, Deyang), middle-GRP areas (Neijiang, Suining) and low-GRP areas (Guangyuan, Aba). Within each city, we randomly selected four Community health centers (CHCs) from urban areas and four township hospitals from rural areas. The CHCs and township hospitals are the main primary care service providers in China [22]. Finally, no less than 6 physicians and 12 patients were randomly selected from each practice. This study differed from the original QUALICOPC protocol, which surveyed nine consecutive patients for each general practitioner [18]. According to national statistics, in 2016, the average daily number of visits per primary care institution was 9.8 in Sichuan province and 12.9 in China [23]. There were often far less than nine patients for each physician per day, especially in township hospitals. Given this concern, two to three patients per primary physician were recruited in the study.

### Survey instrument

**Fig. 1:**
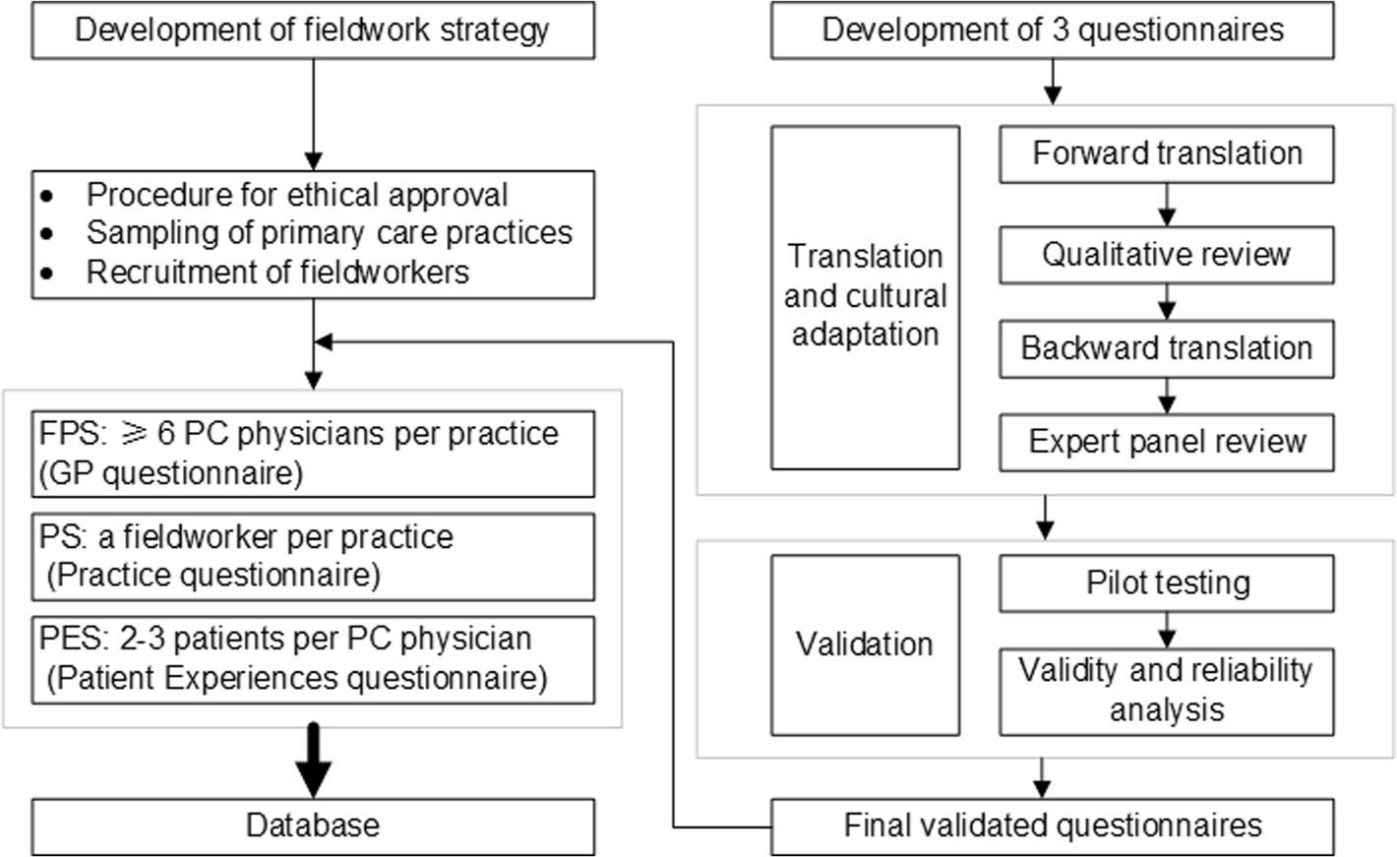
The process of questionnaires adaptation and fieldwork strategy

In the QUALICOPC study framework, there are questionnaires for PC physicians (FPS = the Family Physician Survey), their patients (PES=Patient Experiences Survey and PVS=Patient Values Survey) and fieldworkers (PS=Practice Survey) [18]. The original questionnaires were published elsewhere [19]. We adopted the FPS and PES to analyze physician and patient perceptions of the PC service capacity, and the PS to describe primary care settings. The original questionnaires were translated from English to Chinese through a formal forward-back translation process. We performed a cross-cultural adaptation of QUALICOPC questionnaire for use in China, and added some questions about the background of the primary care providers and patients (e.g., the type of medical insurance). Some questions were excluded, either because their content did not apply to China (e.g., “after a patient has been discharged, how long does it usually take to receive a discharge report from the hospital most frequented by your patients,” because patients usually receive a discharge report at the time of their discharge in China), or because too few variations were observed in the participants’ answers (e.g., place of mother’s birth). After translation and adaptation, we conducted a pilot test to validate and revise the questionnaires, making them more applicable and easier to understand. The process of questionnaire adaptation and fieldwork strategy is illustrated in Fig. 1.

### Data collection

Between October 2017 and February 2018, we implemented the survey for primary care physicians and their patients based on the QUALICOPC study protocol [18, 24]. A total of 319 primary care physicians agreed to participate in the study and completed the FPS. The response rate was 96.4% (*n* = 319/331). For the PES, trained field workers invited patients who had a face-to-face consultation with physicians and who were over the age of 18 years and able to speak and read Chinese. Data were collected at the end of the consultation with the physician. Six hundred forty-one patients agreed and completed the patient experience questionnaire, giving a response rate of 91.4% (*n* = 641/701). This study protocol was approved by the Institutional Review Board (IRB) of West China Hospital in Sichuan University. The surveys were carried out anonymously.

### Statistical analysis

To compare the health service capacity of primary care, we developed capacity indicators (CI) for six PC dimensions: “Accessibility” (ACCS), “Continuity” (CONT), “Comprehensiveness” (COMP), “Coordination” (COOR), “Quality of care” (QUAL), “Equity” (EQ). Each dimension was measured with a set of nominal questions (variables) developed by the QUALICOPC project [11]. The actual questions we used to measure different dimensions are provided in Additional file 1. According to Oleszczyk and Krzton-Krolewiecka’s calculation method, all variables were evaluated by a scale ranging from − 1 (extremely negative) to + 1 (extremely positive). The capacity indicator for each dimension was calculated as an arithmetic mean (μ) of variables. A detailed description of the variable values and data analysis is available in QUALCOPC studies from Poland [17, 25].

Data were entered through the EpiData Software with double entry and validation. Missing data was minimal and was replaced with Mean Imputation (MI). Characteristics of participants were summarized using descriptive statistics. Categorical variables are expressed as proportions. Continuous variables are expressed as means and standard deviations. All analyses were conducted using the Statistical Package for Social Sciences (SPSS) Version 23.0.

## Results

### Characteristics of respondents

Our dataset contained a total 319 PC physician and 641 patient surveys. They were from 48 primary care practices in different level of GDP areas. In the physician sample, the proportion of male and female doctors was similar. The mean age of the participants was 39.4 (SD = 12.1), and their mean years of experience in PC was 17.21 (SD = 12.26). Nearly half of the physicians worked in general practice, the rest in traditional Chinese medicine and other specialties. In the patient sample, the mean age of respondents was 53.1 (SD = 11.3). Only 11.2% had a college education or higher. More than half reported their health status was fair. The detailed socio-demographic characteristics of the study participants is presented in Table 1.

**Table 1:** Socio-demographic characteristics of study participants by GRP area

Characteristic	Total n (%)	High-GRP areas n (%)	Middle-GRP areas n (%)	Low-GRP areas n (%)
Physicians				
Total	319 (100)	107 (33.5)	115 (36.1)	97 (30.4)
Gender				
Male	159 (49.8)	49 (45.8)	57 (49.6)	53 (54.6)
Female	160 (50.2)	58 (54.2)	58 (50.4)	44 (45.4)
Age (years)				
< 30	75 (23.5)	26 (24.3)	32 (27.8)	17 (17.5)
30–	96 (30.1)	36 (33.6)	32 (27.8)	28 (28.9)
40–	89 (27.9)	27 (25.2)	34 (29.6)	28 (28.9)
≥ 50	59 (18.5)	18 (16.8)	17 (14.8)	24 (24.7)
Education				
High school or below	3 (0.9)	1 (0.9)	1 (0.9)	1 (1.0)
Junior college	185 (58.0)	55 (51.4)	74 (64.3)	56 (57.7)
College	131 (41.1)	51 (47.7)	40 (34.8)	40 (41.2)
Practice area				
Urban	136 (42.6)	51 (47.7)	51 (44.3)	34 (35.1)
Rural	183 (57.4)	56 (52.3)	64 (55.7)	63 (64.9)
Experience in PC (years)				
< 5	55 (17.2)	17 (15.9)	24 (20.9)	14 (14.4)
5–	89 (27.9)	35 (32.7)	34 (29.6)	20 (20.6)
15–	91 (28.5)	31 (29.0)	34 (29.6)	26 (26.8)
25–	84 (26.3)	24 (22.4)	23 (20.0)	37 (38.1)
Specialization				
General practice	157 (49.2)	55 (51.4)	52 (45.2)	50 (51.5)
Traditional Chinese medicine	112 (35.1)	35 (32.7)	46 (40.0)	31 (32.0)
Specialist and other	50 (15.7)	17 (15.9)	17 (14.8)	16 (16.5)
Patients				
Total	641 (100)	233 (36.3)	192 (30.0)	216 (33.7)
Gender				
Male	301 (47.0)	105 (45.1)	98 (51.0)	98 (45.4)
Female	340 (53.0)	128 (54.9)	94 (49.0)	118 (54.6)
Age (years)				
18–	56 (8.7)	18 (7.7)	18 (9.4)	20 (9.3)
40–	211 (32.9)	82 (35.2)	49 (25.5)	80 (37.0)
50–	165 (25.7)	65 (27.9)	50 (26.0)	50 (23.1)
≥ 60	209 (32.6)	68 (29.2)	75 (39.1)	66 (30.6)
Education				
Primary school or illiteracy	190 (29.6)	59 (25.3)	61 (31.8)	70 (32.4)
Middle school	190 (29.6)	53 (22.7)	71 (37.0)	66 (30.6)
High school	189 (29.5)	63 (27.0)	52 (27.1)	74 (34.3)
College or higher education	72 (11.2)	58 (24.9)	8 (4.2)	6 (2.8)
Employment status				
Employed	288 (44.9)	115 (49.4)	90 (46.9)	83 (38.4)
Self-employed or family business	223 (34.8)	60 (25.8)	67 (34.9)	96 (44.4)
Retired/unemployed	130 (20.3)	58 (24.9)	35 (18.2)	37 (17.1)
Self-evaluated health status				
Very good	35 (5.5)	9 (3.9)	13 (6.8)	13 (6.0)
Good	136 (21.2)	51 (21.9)	44 (22.9)	41 (19.0)
Fair	346 (54.0)	127 (54.5)	99 (51.6)	120 (55.6)
Poor	124 (19.3)	46 (19.7)	36 (18.8)	42 (19.4)
Declared household income				
Below average	326 (50.9)	119 (51.1)	100 (52.1)	107 (49.5)
Average	315 (49.1)	114 (48.9)	92 (47.9)	109 (50.5)
Above average	0 (0)	0 (0)	0 (0)	0 (0)
Chronic disease				
Yes	428 (66.8)	164 (70.4)	131 (68.2)	133 (61.6)
No	213 (33.2)	69 (29.6)	61 (31.8)	83 (38.4)

Note: *GRP* Gross Regional Product, *PC* Primary Care

As for the general characteristics of the practices, the 48 primary care practices were located almost equally between urban and rural areas. Few primary care institutions indicated clear information on opening hours (25.0%) and how to get out-of-hours care (20.8%). In practices that are not on the ground floor, 19 practices (39.58%) didn’t provide an elevator. Almost all (95.8%) practices had no handicap adjusted toilet. Only 31.2% of practices were easily accessible for patients using a wheelchair or a stroller.

### Perceived capacity of primary health care service

The mean values and standard deviations of each dimension of the capacity index are presented in Table 2. From the perspective of primary care physicians, the dimensions ranging from best to worst according to the scores were equity (0.63), accessibility (0.55), quality (0.47), continuity (0.40), comprehensiveness (0.34), and coordination (0.01). In the equity dimension (the best), the majority (87.8%) of the physicians reported that there were no restrictions when accepting new patients. Almost half of the physicians almost always provided health care to people even if they were not remunerated. In the coordination dimension (the worst), only 25.1% always or usually received new patients’ medical records from the previous doctor. The detailed questions and responses on equity and coordination dimension are given in Additional file 2.

**Table 2:** Capacity indexes of the core dimensions in primary care in China

Item	Physician		Patient	
	$\overline{\mathrm{x}}$	SD	$\overline{\mathrm{x}}$	SD
Accessibility	0.55	0.20	0.20	0.12
Continuity	0.40	0.16	0.31	0.33
Coordination	0.01	0.14	0.17	0.27
Comprehensiveness	0.34	0.09	0.53	0.26
Quality	0.47	0.37	0.73	0.20
Equity	0.63	0.20	0.52	0.26

Note: x- mean values, SD- standard deviation

Calculation method: all variables were evaluated by a scale ranging from −1 (extremely negative) to + 1 (extremely positive). The capacity indicator for each dimension was calculated as an arithmetic mean (μ) of variables

From the patient perspective, the dimensions ranging from best to worst according to the scores were quality (0.73), comprehensiveness (0.53), equity (0.52), continuity (0.31), accessibility (0.20), and coordination (0.17). In the quality dimension (the best), over 90% of patients felt that their doctors were polite and listened carefully, and the vast majority of patients (96.4%) were willing to recommend their doctors to friends or relatives. In the coordination dimension (the worst), only a few patients (5.5%) reported that their GP informed the medical specialist about their illness when they were referred, and 5.6% thought their GP knew the results after treatment by a medical specialist. The detailed questions and responses on the quality and cooperation dimensions are given in Additional file 2.

**Fig. 2:**
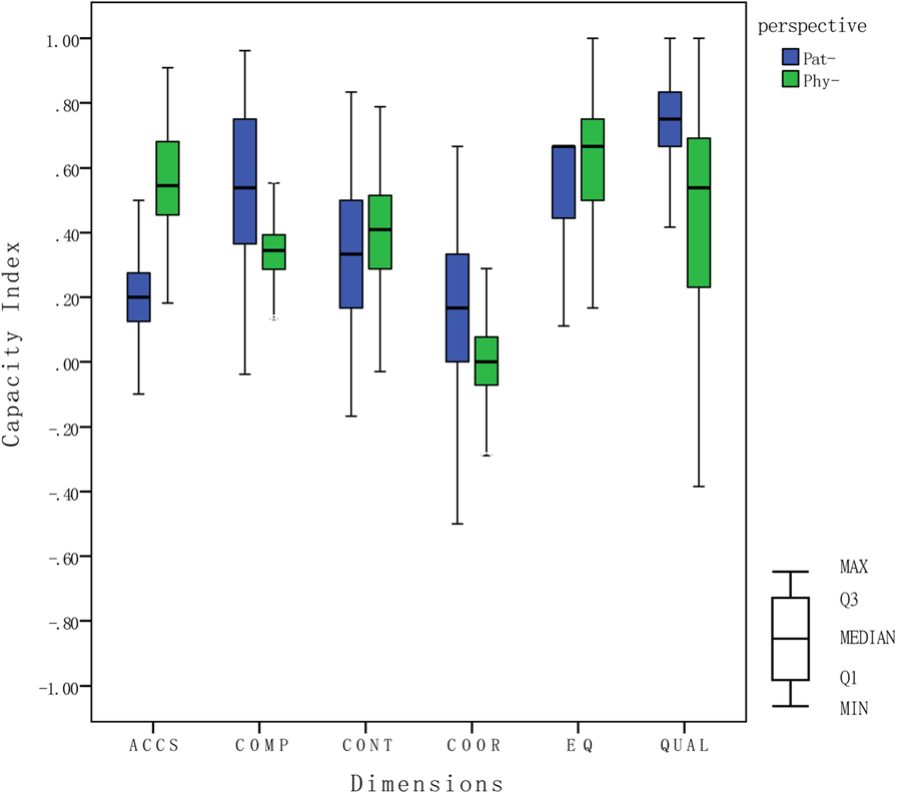
Perception of the core dimensions in primary care from physicians and patients. Q1-the first quartile, Q3-the third quartile, MIN-the minimum, MAX-the maximum; ACCS-Accessibility, COMP-Comprehensiveness, CONT-Continuity, COOR-Coordination, EQ- Equity, QUAL- Quality; Pat- = Patient; Phy- = Physician

**Fig. 3:**
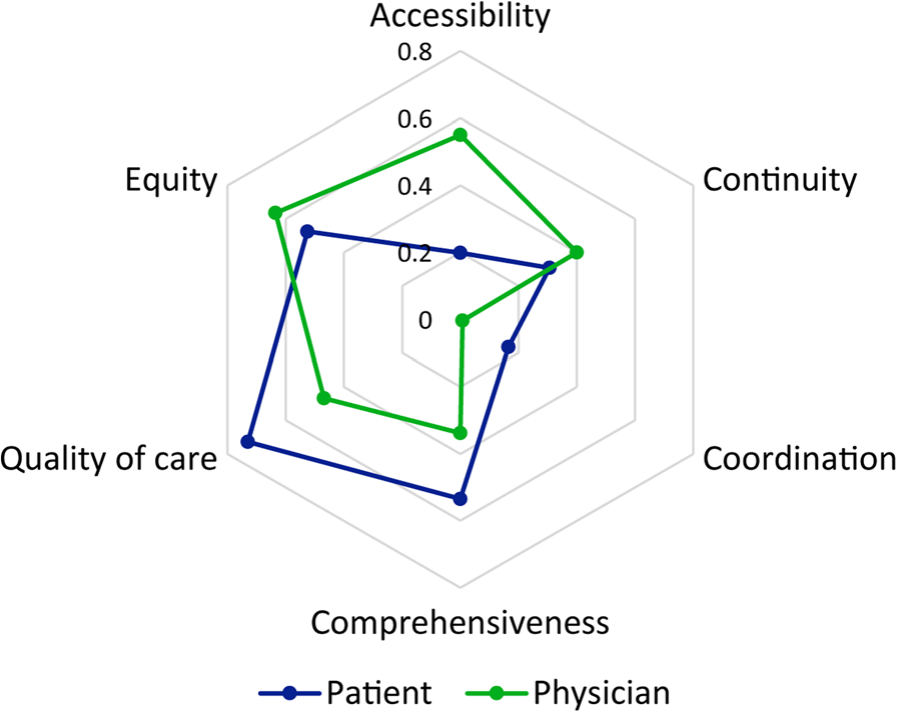
Differences in the perspectives of physicians and patients

Figures 2 and 3 present distributions of capacity indexes for the core dimensions in primary care and show the differences in perspectives of physicians and patients about the primary care health service capacity. Physicians reported higher scores than patients in the accessibility, continuity, and equity dimensions. In contrast, patients reported higher scores in the coordination, comprehensiveness, and quality dimensions than physicians. Both physicians and patients perceived that coordination is the weakest dimension of the primary care service. The largest gap in perceived capacity index between physicians and patients was in the accessibility dimension. 24.8% of the patients thought the practice was too far away from their living or working places and nearly one-third of patients usually take more 20 min to travel from their home to the practice. The detailed information of patients on the accessibility dimension are given in Additional file 2.

## Discussion

To the best of our knowledge, this is the first study to measure the primary care service delivery using the QUALICOPC survey in China, and this is also the first study to compare both the perspectives of physicians and patients by QUALICOPC in the world. Our study showed differences and similarities in the core dimensions of the primary care service capacity between physicians and patients in west China.

Firstly, the perception of the best dimension was different among the physicians and the patients. From the point of view of PC physicians, equity of care scored the best. One explanation for such a finding could be that most PC physicians treated their patients fairly, e.g., had no restrictions to accepting new patients, and prescribed the cheapest equivalent medicine to reduce financial obstacles to disadvantaged patients. An alternative explanation for this finding is that the insurance coverage expansion promoted equity in economic access to primary care [26]. In our study, only a few patients (29, 4.5%) postponed or abstained from a visit to a physician because of lack of insurance. Previous studies revealed that medical insurance may have played an important role in health equity [27, 28]. Quality of service ranks highest among the dimensions in the perspective of patients. This is inconsistent with a previous study that found the Chinese primary health care system is poor in quality [5]. This difference in findings is probably because the other study focused on outcome measures, while the variables in our study are mainly process measures (e.g., communication, careful treatment).

In addition, physicians had higher perceptions of accessibility, while patients’ experiences with accessibility seemed to be relatively worse. In general, accessibility was perceived well in terms of access to services, e.g., making an appointment for a visit and waiting time for consultation in primary care. Our finding that there was a barrier to spatial accessibility of primary care is consistent with literature on this topic [29]. Poor accessibility scores for patients also may relate to the practice characteristics; for example, few primary care institutions outside provided clear information on when open and how to get out-of-hours care.

Lastly, coordination ranked the lowest among the core dimensions from the perspective of both physicians and patients. Similarly, Polish GPs and patients had lower perceptions of coordination [17, 25]. The lack of efficient information flow could be the explanation of worse evaluation of coordination of care. The results showed that medical records were seldomly provided by the previous doctor and results after treatment were often not known when patients transferred. Findings of recent studies indicated that the coordination dimension was more related to the dissemination of information among family physicians or between family physicians and specialists (primary and secondary care) [25, 30]. Small private practices which may lead to “a culture of individualism” could also impede coordination of care [31]. An alternative interpretation is that the coordination of care is relevant to planners of PC and the opportunities offered by health managers in the local community [32].

In China, poor coordination may be explained by “isolated” and “fragmented” healthcare services, for example, primary healthcare centers and hospitals operate independently and compete for patients [33, 34]. Integrated care has been suggested as one strategy for promoting coordinated healthcare delivery. In 2016, the report on the Deepening Health Reform in China proposed a hierarchical medical system in accordance with a people-centered integrated care model for strengthening health care [35]. However, the implementation of this policy has not brought about as many improvements as expected. The referral rate in China was far lower than the general referral rate (20–30%) published by the World Health Organization [36]. The previous studies indicated that this may relate to the lack of coordination and continuity between hospitals at the different levels [6, 37, 38]. We suggest that policy makers should focus more on the coordination dimension of primary care when enacting heath policy reforms. Sharing medical information (e.g., electronic medical records), shared management (e.g., collaboration skills), and payment stimulus could be the suggestions to promote the coordination [39–41].

Our study has several limitations. Although the questionnaires were designed and validated for an international study and our material allows for international comparisons, the questions were not specifically designed to map the context of China. Thus, for Chinese circumstances, we removed or added some items, and made some adaptations in the original questionnaires. These may lead to a bias in the comparability of our findings with international results. Another limitation is that all information was based on the physician and patient self-reported data. Answers are subjective and could be under- or over-reported, and, therefore, could be inaccurate. Recall bias may also apply. Due to limited clinical knowledge, it is not possible to assess certain aspects of technical quality from the patient’s perception. In addition, further research needs to expand the sample size to more primary care settings or regions in China to increase generalizability of findings.

## Conclusion

This study provided an evaluation of the service capacity of primary health care in one province in China using the QUALICOPC protocol. Although primary health care reform may have been effective in helping patients acquire better primary care services, our results suggest that coordination is still perceived to be problematic for both physicians and patients. Improving the coordination of care has to be one of the main goals in the future primary care reforms in China. More efforts are needed to improve the coordinated relationship among primary care physicians and between primary and secondary care physicians. Lessons from primary care reform based on this study can also serve as reminder for other low-income and middle-income countries undertaking similar endeavors in the future. In addition, we have demonstrated that one method of comparing multi-stakeholder’s perceptions may help effectively to assess primary care service capacity.

## Additional files

Additional file 1: Questions of GP questionnaire and Patient Experiences questionnaire in the core dimensions. (DOCX 27 kb)

Additional file 2: Responses of primary care physicians and their patients. (DOCX 38 kb)

## Abbreviations

CHCs: Community health centers
FPS: the Family Physician Survey
GRP: Gross Regional Product
MI: Mean Imputation
PC: Primary care
PCAT: Primary Care Assessment Tool
PES: Patient Experiences Survey
PS: Practice Survey
PVS: Patient Values Survey
QUALICOPC: Quality and Costs of Primary Care
