# Supplementary material for: The health service capacity of primary health care in West China: different perspectives of physicians and their patients
Source: BMC Health Serv Res. 2019 Feb 28;19:143. doi: 10.1186/s12913-019-3964-x (PMC6396462; doi:10.1186/s12913-019-3964-x)
Supplement: Supplementary file 1 — Questions of GP questionnaire and Patient Experiences questionnaire in the core dimensions. (DOCX 27 kb) [file 12913_2019_3964_MOESM1_ESM.docx]

**Additional file 1**

**Questions of GP questionnaire and Patient Experiences questionnaire in the core dimensions**

| **Dimension** | **Question (GP questionnaire)** | **Question (Patient Experiences questionnaire)** |
| --- | --- | --- |
| ACCS | ▶What is the distance by road from your (main) practice building to:  -The nearest GP practice  -The nearest consultant/outpatient clinic  -The nearest general or university hospital  ▶How many hours on average working day is your practice open for patient care (lunch breaks excluded)  ▶Is it possible for your patients to visit your practice/center:  - After 18.00h (at least once per week)  - On a weekend day (at least once per month)  ▶What percentage of your patient consultation is by appointment?  ▶▶In the past 12 months, have you ever done the following to reduce financial obstacles to disadvantaged patients:  - Provide free samples of medication  - Prescribe the cheapest equivalent medicine  - Not charge the patient  ▶▶In the past 12 months, how often have you noticed that patients delayed their visits for financial reasons? | ▶ The doctor took sufficient time?  ▶Think about the practice that you visited today. Do you agree with the following?  -The opening hours are too restricted  -If I need a home visit, I can get one  -The practice is too far away from where I am living or working  -When I called this practice, I had to wait too long to speak to someone  -I know how to get evening, night and weekend services  ▶How long does it usually take you to travel from your home to this practice  ▶Did you make an appointment for this visit to your doctor?  ▶Was it easy to get the appointment?  ▶How many days did you wait for this visit?  ▶How long did you wait today between arriving in the practice and the consultation?  ▶Do you think it is too difficult to see a GP during evening, nights and weekends?  ▶▶It is difficult to get a referral to a medical specialist from my GP?  ▶Why did you go to the emergency department instead of going to a GP? |
| CONT | ▶▶Do you use clinical guidelines for the treatment of the following? (Chronic heart failure; Asthma; COPD; Diabetes)  ▶▶▶In the past 12 months, have you been involved in a disease management program for patient with the following chronic conditions? (such program are multidisciplinary approaches across practices, often based on protocols) (Chronic heart failure; Asthma; COPD; Diabetes; high blood pressure)  ▶▶ In the past 12 months, has the following occurred in your practice/center?  -Feedback on your prescriptions or referrals by health authority or insurer?  -Feedback from colleague GPs (peer review or practice visitation)?  -Investigation into the satisfaction of your patients?  ▶▶In case of referral, who usually decides about where the patient is referred to?  ▶▶In case of referral, to what extent do you take into account the following considerations  - The patient's preference where to go  - The travel distance for the patient  - Your previous experiences with the medical specialist  - Comparative performance information on medical specialists  - Waiting time for the patient  - Costs for the patient  ▶▶If new patients enter your practice, do you receive their medical records from their previous doctor?  ▶How do you keep patient medical records?  ▶In the past 2 years, have you used your medical record system to list a selection of patients on the basis of age, diagnosis or risk?  ▶▶For which of the following purposes do you use a computer in your practice?  -Not applicable (I don’t use a computer)  -Making appointments  -Issuing drug prescriptions  -Keeping records of consultations  -Sending referral letters to medical specialists  -Storing diagnostic test results  -Searching medical information on the internet  -Sending prescriptions to the pharmacy | ▶ The doctor had my medical records at hand?  ▶He/she knows important information about my medical background  ▶He/she knows about my living situation  ▶In the past 2 years, has a GP from this practice ever asked you about all the medications you take (also those prescribed by other doctors)?  ▶▶If I visit another GP besides my own GP, he/she has the necessary information about me  ▶▶After treatment by a medical specialists, my GP knows the results |
| COMP | ▶Please tick the equipment used in your practice by yourself or your staff:  ▶How do you have access to laboratory facilities?  ▶How do you have access to X-ray facilities?  ▶To what extent are you involved in the treatment and follow-up of patients in your practice population with the following diagnoses  ▶To what extent are the following activities carried out in your practice population by you (or by your staff) and not by a medical specialist?  ▶When do you, or your staff, measure blood pressure?  ▶When do you, or your staff, measure blood cholesterol level?  ▶To what extent are you involved in health education as regards the following topics: (Smoking; Diet; Problematic use of alcohol; Physical exercise)  ▶Are you or your practice staff involved in the following activities?  -Routine antenatal care  -Immunization of children (as part of a program)  -Pediatric surveillance of children under 4 years  -Influenza vaccination (as part of a program)  -Palliative care  ▶During the past 12 months, have you offered (a) special session(s) or clinics for the following groups? (Diabetic patients; Hypertensive patients; Pregnant women; Elderly)  ▶If you confronted through your patients contacts with the following occurrences, would you report this (for instance to an authority)  -Repeated accidents in an industrial setting  -Frequent respiratory problems in patients living near a certain industry  -Repeated cases of food poisoning among people living in a certain district | ▶In the past 12 months, has a GP from this practice talked to you about how to stay healthy? (For instance, about diet, alcohol or smoking)  ▶Would most people visit a GP for the following?  -Cut finger that needs to be stitched?  -Removal of a wart  -Routine health checks  -Deteriorated vision  -Help to quit smoking  -A child with a severe cough  -Stomach pain  -Blood in the stool  -Sprained ankle  -Anxiety  -Domestic violence  -Sexual problems  -Relationship problems  -Advice for choosing the best hospital or specialist for a certain treatment |
| COOR | ▶Which of the following disciplines are working in your practice/center? (Receptionist/medical secretary; Practice nurse; Community/home care nurse; Assistant for laboratory work; Manager of the center or practice; Midwife; Physiotherapist; Dentist; Pharmacist; Social worker)  ▶▶▶In the past 12 months, have you been involved in a disease management program for patient with the following chronic conditions? (such program are multidisciplinary approaches across practices, often based on protocols) (Chronic heart failure; Asthma; COPD; Diabetes; high blood pressure)  ▶▶In case of referral, who usually decides about where the patient is referred to?  ▶▶In case of referral, to what extent do you take into account the following considerations  - The patient's preference where to go  - The travel distance for the patient  - Your previous experiences with the medical specialist  - Comparative performance information on medical specialists  - Waiting time for the patient  - Costs for the patient  ▶▶If new patients enter your practice, do you receive their medical records from their previous doctor?  ▶▶For which of the following purposes do you use a computer in your practice?  -Not applicable (I don’t use a computer)  -Making appointments  -Issuing drug prescriptions  -Keeping records of consultations  -Sending referral letters to medical specialists  -Storing diagnostic test results  -Searching medical information on the internet  -Sending prescriptions to the pharmacy  ▶How often do you meet face-to-face with the following professionals (either professionally or socially): (Other GP; Practice nurse; Ambulatory medical specialist; Hospital medical specialist; Pharmacist; Home care nurse; Midwife; Physiotherapist; Social worker; Dietician)  ▶How often do you ask advice (e.g. by telephone) from the following medical specialists?  ▶To what extent do you use referral letters (including details on provisional diagnosis and possible test results) when you refer patients to a medical specialist?  ▶To what extent do medical specialists inform you after they have finished the treatment or diagnostics of your patients | ▶I thought tests or examinations were repeated unnecessarily  ▶▶If I visit another GP besides my own GP, he/she has the necessary information about me  ▶When I am referred, my GP informs the medical specialist about my illness  ▶▶After treatment by a medical specialist, my GP knows the results  ▶▶It is difficult to get a referral to a medical specialist from my GP?  ▶In the past 12 months, have you been examined or treated by a nurse at your GP's practice? |
| QUAL | ▶▶Do you use clinical guidelines for the treatment of the following? (Chronic heart failure; Asthma; COPD; Diabetes)  ▶▶▶In the past 12 months, have you been involved in a disease management program for patient with the following chronic conditions? (such program are multidisciplinary approaches across practices, often based on protocols) (Chronic heart failure; Asthma; COPD; Diabetes; high blood pressure)  ▶▶ In the past 12 months, has the following occurred in your practice/center?  -Feedback on your prescriptions or referrals by health authority or insurer?  -Feedback from colleague GPs (peer review or practice visitation)?  -Investigation into the satisfaction of your patients? | ▶The doctor was polite?  ▶The doctor listened carefully to me?  ▶The doctor hardly looked at me when we talked?  ▶The doctor asked question about my health problem?  ▶I couldn't really understand what the doctor was trying to explain  I could recommend this doctor to a friend or relative?  ▶The doctor asked about possible other problems besides the one I just came for?  ▶This doctor doesn't just deal with medical problems but can also help with personal problems and worries?  ▶After this visit, I feel I can cope better with my health problem/illness than before?  ▶I thought I got the wrong medication or wrong dose?  ▶I thought I got incorrect results of a test or X-ray?  ▶If you are unhappy with treatment you received, do you think this doctor would be prepared to discuss it with you? |
| EQ | ▶▶In the past 12 months, have you ever done the following to reduce financial obstacles to disadvantaged patients:  - Provide free samples of medication  - Prescribe the cheapest equivalent medicine  - Not charge the patient  ▶▶In the past 12 months, how often have you noticed that patients delayed their visits for financial reasons?  ▶Which restrictions do you apply to accepting new patients?  Do you provide health care to people, when you are not ▶remunerated for this (for instance uninsured, illegal immigrants)? | ▶In the past 12 months, has one of the following happened to you in this practice?  -the doctor or staff acted negatively to you  -other patients were treated better than you  -the doctor was too much concerned about money  -the doctor or staff showed disrespect because of your ethnic background  -the doctor or staff showed disrespect because of your gender  ▶In the past 12 months, did you postpone or abstain from a visit to this doctor or another doctor GP when you needed one?  ▶What was the most important reason why you did not visit a GP? |

Note: ACCS= Accessibility, CONT= Continuity, COMP= Comprehensiveness, COOR=Coordination, QUAL= Quality of care, EQ= Equity

We marked some questions in a variety of colors which means they can measure the dimensions corresponding to the colors. For example, ACCS and EQ is measured by the same question “In the past 12 months, have you ever done the following to reduce financial obstacles to disadvantaged patients”, we marked this question in two colors: blue and red.
