# Supplementary material for: The health service capacity of primary health care in West China: different perspectives of physicians and their patients
Source: BMC Health Serv Res. 2019 Feb 28;19:143. doi: 10.1186/s12913-019-3964-x (PMC6396462; doi:10.1186/s12913-019-3964-x)
Supplement: Supplementary file 2 — Responses of primary care physicians and their patients. (DOCX 38 kb) [file 12913_2019_3964_MOESM2_ESM.docx]

**Additional file 2**

1.1 Responses of primary care physicians on Equity (the best) and Coordination (the worst) dimension

| Dimension | Questions | Response | | |
| --- | --- | --- | --- | --- |
|  |  | Answers | N | Frequency (%) |
| EQ | ▶In the past 12 months, have you ever done the following to reduce financial obstacles to disadvantaged patients:(Yes/No) | Provide free samples of medication (Yes) | 166 | 52.0 |
|  |  | Prescribe the cheapest equivalent medicine (Yes) | 315 | 98.7 |
|  |  | Not charge the patient (e.g. for co-payments) (Yes) | 150 | 47.0 |
|  | ▶In the past 12 months, how often have you noticed that patients delayed their visits for financial reasons? | Frequently | 43 | 13.5 |
|  |  | Occasionally | 160 | 50.2 |
|  |  | Never | 116 | 36.4 |
|  | ▶Which restrictions do you apply to accepting new patients? (More than one answer possible) | No restrictions (everyone is accepted) | 280 | 87.8 |
|  |  | No new patients are taken above a maximum number | 18 | 5.6 |
|  |  | No new patients are taken above a certain age | 15 | 4.7 |
|  |  | No new patients are taken outside my geographical working area | 4 | 1.3 |
|  |  | I use a wait period for new patients | 0 | 0.0 |
|  |  | Acceptance depends on patients' medical history | 14 | 4.4 |
|  |  | Acceptance depends on patients' insurance status | 1 | 0.3 |
|  | ▶Do you provide health care to people, when you are not remunerated for this (for instance uninsured, illegal immigrants)? | Yes, (almost always) | 147 | 46.1 |
|  |  | Yes, but only in urgent cases | 111 | 34.8 |
|  |  | Yes, sometimes | 60 | 18.8 |
|  |  | No | 1 | 0.3 |
| COOR | ▶Which of the following disciplines are working in your practice/center? | Receptionist/medical secretary | 112 | 35.1 |
|  |  | Practice nurse | 79 | 24.8 |
|  |  | Community/home care nurse | 113 | 35.4 |
|  |  | Assistant for laboratory work | 177 | 55.5 |
|  |  | Manager of the center or practice (not a physician) | 116 | 36.4 |
|  |  | Midwife | 71 | 22.3 |
|  |  | Physiotherapist | 169 | 53.0 |
|  |  | Dentist | 138 | 43.3 |
|  |  | Pharmacist | 190 | 59.6 |
|  |  | Social worker | 34 | 10.7 |
|  | ▶In the past 12 months, have you been involved in a disease management program for patient with the following chronic conditions? (such program are multidisciplinary approaches across practices, often based on protocols) (Yes/No) | Chronic heart failure (Yes) | 180 | 56.4 |
|  |  | Asthma (Yes) | 183 | 57.4 |
|  |  | COPD (Yes) | 231 | 72.4 |
|  |  | Diabetes (Yes) | 307 | 96.2 |
|  |  | high blood pressure (Yes) | 307 | 96.2 |
|  | ▶In case of referral, who usually decides about where the patient is referred to? | I do | 19 | 6.0 |
|  |  | The patient does | 34 | 10.7 |
|  |  | It is shared decision | 266 | 83.4 |
|  | ▶If new patients enter your practice, do you receive their medical records from their previous doctor? | Yes, always or usually | 80 | 25.1 |
|  |  | Only occasionally | 142 | 44.5 |
|  |  | Rarely | 83 | 26.0 |
|  |  | Never | 14 | 4.4 |
|  | ▶For which of the following purposes do you use a computer in your practice | Not applicable (I don’t use a computer) | 0 | 0.0 |
|  |  | Making appointments | 70 | 21.9 |
|  |  | Issuing drug prescriptions | 313 | 98.1 |
|  |  | Keeping records of consultations | 313 | 98.1 |
|  |  | Sending referral letters to medical specialists | 72 | 22.6 |
|  |  | Storing diagnostic test results | 237 | 74.3 |
|  |  | Searching medical information on the internet | 226 | 70.8 |
|  |  | Sending prescriptions to the pharmacy | 311 | 97.5 |
|  | ▶To what extent do you use referral letters (including details on provisional diagnosis and possible test results) when you refer patients to a medical specialist? I use letters: | for all patients that I refer | 184 | 57.7 |
|  |  | for most patients that I refer | 55 | 17.2 |
|  |  | for a minority of patients that I refer | 57 | 17.9 |
|  |  | seldom or never | 23 | 7.2 |
|  | ▶To what extent do medical specialists inform you after they have finished the treatment or diagnostics of your patients | (Almost) always | 38 | 11.9 |
|  |  | Usually | 53 | 16.6 |
|  |  | Occasionally | 116 | 36.4 |
|  |  | Seldom or Never | 112 | 35.1 |

1.2 Responses of primary care physicians on Equity (the best) and Coordination (the worst) dimension

| Dimension | Questions | Response | | |
| --- | --- | --- | --- | --- |
| COOR | ▶In case of referral, to what extent do you take into account the following considerations | Always  (n, %) | Sometimes  (n, %) | Never  (n, %) |
|  | The patient's preference where to go | 177 (55.5) | 133 (41.7) | 9 (2.8) |
|  | The travel distance for the patient | 199 (62.4) | 112 (35.1) | 8 (2.5) |
|  | Your previous experiences with the medical specialist | 105 (32.9) | 147 (46.1) | 67 (21.0) |
|  | Comparative performance information on medical specialists | 196 (61.4) | 105 (32.9) | 18 (5.6) |
|  | Waiting time for the patient | 191 (59.9) | 115 (36.1) | 13 (4.1) |
|  | Costs for the patient | 150 (47.0) | 153 (48.0) | 16 (5.0) |
|  | ▶How often do you meet face-to-face with the following professionals (either professionally or socially): | Seldom or never  (n, %) | Every 1-3 months  (n, %) | More than once a month  (n, %) |
|  | Other GP | 64 (20.1) | 118 (37.0) | 137 (42.9) |
|  | Practice nurse | 20 (6.3) | 84 (26.3) | 215 (67.4) |
|  | Ambulatory medical specialist | 131 (41.1) | 86 (27.0) | 102 (32.0) |
|  | Hospital medical specialist | 106 (33.2) | 96 (30.1) | 117 (36.7) |
|  | Pharmacist | 39 (12.2) | 82 (25.7) | 198 (62.1) |
|  | Home care nurse | 193 (60.5) | 71 (22.3) | 55 (17.2) |
|  | Midwife | 266 (83.4) | 41 (12.9) | 12 (3.8) |
|  | Physiotherapist | 126 (39.5) | 73 (22.9) | 120 (37.6) |
|  | Social worker | 278 (87.1) | 31 (9.7) | 10 (3.1) |
|  | Dietician | 272 (85.3) | 28 (8.8) | 19 (6.0) |
|  | ▶How often do you ask advice (e.g. by telephone) from the following medical specialists? | Seldom or never  (n, %) | Every 1-3 months  (n, %) | More than once a month  (n, %) |
|  | Pediatrician | 147 (46.1) | 126 (39.5) | 46 (14.4) |
|  | Internist | 134 (42.0) | 134 (42.0) | 134 (42.0) |
|  | Gynecologist | 228 (71.5) | 63 (19.7) | 28 (8.8) |
|  | Surgeon | 221 (69.3) | 63 (19.7) | 35 (11.0) |
|  | Neurologist | 231 (72.4) | 70 (21.9) | 18 (5.6) |
|  | Dermatologist | 238 (74.6) | 63 (19.7) | 18 (5.6) |
|  | Geriatrician | 227 (71.2) | 62 (19.4) | 30 (9.4) |
|  | Psychiatrist mental health professional | 244 (76.5) | 52 (16.3) | 23 (7.2) |
|  | Radiologist | 230 (72.1) | 47 (14.7) | 42 (13.2) |

Note: EQ- Equity, COOR-Coordination

2 Responses of patients on Quality (the best) and Coordination (the worst) dimension

| Dimension | Questions | Responses | | |
| --- | --- | --- | --- | --- |
|  |  | Yes  n (%) | No  n (%) | Don't know  n (%) |
| QUAL | The doctor was polite | 587 (91.6) | 54 (8.4) | - |
|  | The doctor listened carefully to me | 588 (91.7) | 53 (8.3) | - |
|  | The doctor hardly looked at me when we talked | 176 (27.5) | 465 (72.5) | - |
|  | The doctor asked question about my health problem | 641 (100.0) | 0 (0.0) | - |
|  | I couldn't really understand what the doctor was trying to explain | 350 (54.6) | 291 (45.4) | - |
|  | I could recommend this doctor to a friend or relative | 618 (96.4) | 23 (3.6) | - |
|  | The doctor asked about possible other problems besides the one I just came for | 591 (92.2) | 50 (7.8) | - |
|  | This doctor doesn't just deal with medical problems but can also help with personal problems and worries | 502 (78.3) | 38 (5.9) | 101 (15.8) |
|  | After this visit, I feel I can cope better with my health problem/illness than before | 525 (81.9) | 116 (18.1) | 0 (0.0) |
|  | I thought I got the wrong medication or wrong dose | 3 (0.5) | 612 (95.5) | 26 (4.1) |
|  | I thought I got incorrect results of a test or X-ray | 2 (0.3) | 613 (95.6) | 26 (4.1) |
|  | If you are unhappy with treatment you received, do you think this doctor would be prepared to discuss it with you? | 542 (84.6) | 97 (15.1) | 2 (0.3) |
| COOR | I thought tests or examinations were repeated unnecessarily | 68 (10.6) | 536 (83.6) | 37 (5.8) |
|  | If I visit another GP besides my own GP, he/she has the necessary information about me | 370 (57.7) | 162 (25.3) | 109 (17.0) |
|  | When I am referred, my GP informs the medical specialist about my illness | 35 (5.5) | 51 (8.0) | 372 (58.0) |
|  | After treatment by a medical specialist, my GP knows the results | 36 (5.6) | 93 (14.5) | 381 (59.4) |
|  | It is difficult to get a referral to a medical specialist from my GP | 112 (17.5) | 59 (9.2) | 419 (65.4) |
|  | In the past 12 months, have you been examined or treated by a nurse at your GP's practice? | 351 (54.8) | 263 (41.0) | 27 (4.2) |

Note: QUAL- Quality, COOR-Coordination

3 Responses of patients on accessibility dimension

| Question | Response | | |
| --- | --- | --- | --- |
|  | Yes n (%) | No n (%) | don't know n (%) |
| The doctor took sufficient time | 543 (84.7) | 98 (15.3) | - |
| The opening hours are too restricted | 55 (8.6) | 493 (76.9) | 93 (14.5) |
| If I need a home visit, I can get one | 70 (10.9) | 452 (70.5) | 119 (18.6) |
| The practice is too far away from where I am living or working | 159 (24.8) | 482 (75.2) | 0 (0.0) |
| When I called this practice, I had to wait too long to speak to someone | 46 (7.2) | 103 (16.1) | 492 (76.8) |
| I know how to get evening, night and weekend services | 183 (28.5) | 239 (37.3) | 219 (34.2) |
| Did you make an appointment for this visit to your doctor | 21 (3.3) | 620 (96.7) | - |
| Was it easy to get the appointment? | 21 (3.3) | 0 (0.0) | - |
| Do you think it is too difficult to see a GP during evening, nights and weekends? | 43 (6.7) | 463 (72.2) | 135 (21.1) |
| ▶How long does it usually take you to travel from your home to this practice | n | % |  |
| Less than 20 minutes | 444 | 69.3 |  |
| 20-40 minutes | 130 | 20.3 |  |
| 40-60 minutes | 34 | 5.3 |  |
| More than 1 hour | 33 | 5.1 |  |
| Don't know | 0 | 0.0 |  |
| ▶How many days did you wait for this visit | n | % |  |
| I made the appointment earlier today | 8 | 1.2 |  |
| I made the appointment yesterday | 13 | 2.0 |  |
| I waited 2-7 days | 0 | 0.0 |  |
| I waited more than a week | 0 | 0.0 |  |
| Don't know | 0 | 0.0 |  |
| ▶How long did you wait today between arriving in the practice and the consultation? | n | % |  |
| Less than 15 minutes | 630 | 98.3 |  |
| 15-30 minutes | 11 | 1.7 |  |
| 30-45 minutes | 0 | 0.0 |  |
| 45-60 minutes | 0 | 0.0 |  |
| More than an hour | 0 | 0.0 |  |
| Don't know | 0 | 0.0 |  |
| ▶Why did you go to the emergency department instead of going to a GP? | n | % |  |
| I had something GPs do not treat | 28 | 4.4 |  |
| There was no GP available | 84 | 13.1 |  |
| For financial reasons | 73 | 11.4 |  |
| At the emergency department, I expected a shorter waiting time | 69 | 10.8 |  |
| The emergency department provides better care | 88 | 13.7 |  |
| The emergency department is more convenient to reach | 84 | 13.1 |  |
| Other reason(s) | 0 | 0.0 |  |
